# Protective Effect of *Bauhinia purpurea* on Gentamicin-induced Nephrotoxicity in Rats

**DOI:** 10.4103/0250-474X.58196

**Published:** 2009

**Authors:** B. V. S. Lakshmi, N. Neelima, N. Kasthuri, V. Umarani, M. Sudhakar

**Affiliations:** Malla Reddy College of Pharmacy, Dhulapally, Secunderabad-500 014, India

**Keywords:** *Bauhinia purpurea*, gentamicin, nephrotoxicity, fruit juice and ethanol extracts

## Abstract

The present study was undertaken to evaluate the ethanol extract of leaves of *Bauhinia purpurea* and unripe pods of *Bauhinia purpurea* for its protective effects on gentamicin-induced nephrotoxicity in rats. Nephrotoxicity was induced in Wistar rats by intraperitoneal administration of gentamicin 100 mg/kg/d for eight days. Effect of concurrent administration of ethanol extract of leaves of *Bauhinia purpurea* and unripe pods of *Bauhinia purpurea* at a dose of 300 mg/kg/d given by oral route was determined using serum creatinine, serum uric acid, blood urea nitrogen and serum urea as indicators of kidney damage. The study groups contained six rats in each group. It was observed that the ethanol extract of leaves of *Bauhinia purpurea* and unripe pods of *Bauhinia purpurea* significantly protect rat kidneys from gentamicin-induced histopathological changes. Gentamicin-induced glomerular congestion, blood vessel congestion, epithelial desquamation, accumulation of inflammatory cells and necrosis of the kidney cells were found to be reduced in the groups receiving the leaf and unripe pods extract of *Bauhinia purpurea* along with gentamicin. The extracts also normalized the gentamicin-induced increase in serum creatinine, serum uric acid and blood urea nitrogen levels. This is also evidenced by the histopathological studies.

*Bauhinia purpurea* is a flowering plant (Family: Fabaceae). Several species of this plant are known to possess pharmacological activities. Aqueous extract of leaves have antinociceptive, antiinflammatory and antipyretic[1], hypoglycemic[2], antimalarial, antimycobacterial, antifungal and cytotoxic activities[3]. Antioxidant and hepatoprotective activities of *Bauhinia* species have also been reported[4]. Methanol extract obtained from *Bauhinia purpurea* led to the isolation and identification of 6-butyl-3-hydroxy flavone[5]. However, systematic and scientific reports on the investigation of ethanol extract of leaves and unripe pods of *B. purpurea* for its effects on renal function are scarce. In the present study, an effort has been made to evaluate the effects of the ethanol extract of leaves and unripe pods of this plant on gentamicin-induced nephrotoxicity in rats.

*Bauhinia purpurea* leaves and unripe pods were collected from Dhulapally, Rangareddy district, Hyderabad. The plant was authenticated at the department of Botany, Osmania University. A voucher specimen is deposited there for further reference. Leaves and unripe pods were air dried, powdered to 40 mesh and subjected to Soxhlet extraction at 60° with ethanol. The extract was concentrated under reduced pressure. Leaf extract due to its sticky constituency was suspended in 1% Tween-80 and unripe pod extract with 1% gum acacia for oral administration. Chemical tests for carbohydrates, proteins, alkaloids, flavonoids, triterpenes, glycosides and steroids were carried out on the *Bauhinia purpurea* extracts using the standard procedures available in text books[6].

Healthy, male Wistar rats each weighing 150-200 g were used for this study. The rats were housed in polypropylene cages and maintained under standard conditions (12 h light and dark cycles, at 25±3° and 35-60% humidity). Standard pelletized feed and tap water were provided *ad libitum*. The Institutional Animal Ethical Committee of Malla Reddy College of Pharmacy, Hyderabad, with college Reg. No. 1217/a/08/CPCSEA, approved the study.

Twenty-four male Wistar rats were assigned to four groups, group I was the control group, group II was the gentamicin-treated group, group III was the gentamicin-as well as ethanol extract of leaves-treated group (BPLE) and group IV was the gentamicin- as well as ethanol extract of unripe pods of *B. purpurea*-treated group (BPPE). Each group consisted of six rats. The gentamicin-treated group received 100 mg/kg/day gentamicin (Hi Media Laboratories, Mumbai, India) by the intraperitoneal (i.p.) route[7]. Group III received 100 mg/kg/d gentamicin i.p. and 300 mg/kg/d of the BPLE p.o. for eight days and group IV received 100 mg/kg/d gentamicin i.p. and 300 mg/kg/day of the BPPE p.o. for eight days. Rats in the control group were given sterile saline solution i.p. for the same number of days. After dosing on the 8^th^ day, blood samples were collected via cardiac puncture method at the end of these 24 h. The serum was rapidly separated and processed for determination of serum creatinine, serum uric acid, blood urea nitrogen (BUN) and serum urea using commercially available kits of Span Diagnostics Ltd, Hyderabad, India[8]. Changes in kidney weight were recorded. Three rats per group were sacrificed and both kidneys were isolated from each rat[9]. The kidneys were weighed and processed for histopathological examination[10].

The kidneys were sectioned longitudinally in two halves and were kept in 10% neutral formalin solution[11]. Both kidneys were processed and embedded in paraffin wax and sections were taken using a microtome. The sections were stained with hematoxylin and eosin and were observed under a computerized light microscope. The data obtained was analyzed using one-way ANOVA followed by Dunnet's multiple comparison test. *P* < 0.01 was considered significant.

Serum creatinine, serum uric acid, blood urea nitrogen, serum urea and the weights of the kidneys were found to be significantly increased in rats treated with only gentamicin; whereas treatment with the BPLE and BPPE was found to protect the rats from such effects of gentamicin. As shown in Table 1.

**TABLE 1:** PARAMETERS STUDIED FOR THE NEPHROPROTECTIVE ACTIVITY OF ETHANOL EXTRACTS OF *BAUHINIA PURPUREA*

Groups	Serum creatinine (mg/ml)	Serum uric acid (mg/ml)	Blood urea nitrogen (mg/ml)	Serum urea (mg/ml)	Weight of kidney (g)
Control	1.23±0.021	3.52±0.91	19.24±0.95	39.22±3.54	0.89±0.34
Gentamicin	2.94±0.45*	7.7 ±1.09*	45.40±1.24*	97.03±2.98*	1.28±0.92*
Gentamicin+ Leaf extract	2.17±0.51*	4.4±1.54*	9.19±1.54*	64.78±3.12*	1.07±0.23*
Gentamicin+ Unripe pod extract	1.60±0.071*	3.25±2.05*	22.43±2.12*	48.22±2.76*	1.18±0.98*
One-way F	62.98	191.48	2239.58	78.96	4881.66
ANOVA d.f	3, 20	3, 20	3, 20	3, 20	3, 20

Values are expressed as mean±SEM. n=6 rats in each group.

* P<0.01 compared to control group.

Control rats showed normal glomerular and tubular histology (fig. 1A) Group II animals exhibited glomerular, peritubular and blood vessel congestion and result in the presence of inflammatory cells in kidney sections (fig. 1B). Concurrent treatment with the ethanolic extract of leaves (fig. 1C) and unripe pods (fig. 1D) were found to reduce such changes in kidney histology induced by gentamicin (Table 2).

**Fig. 1:**
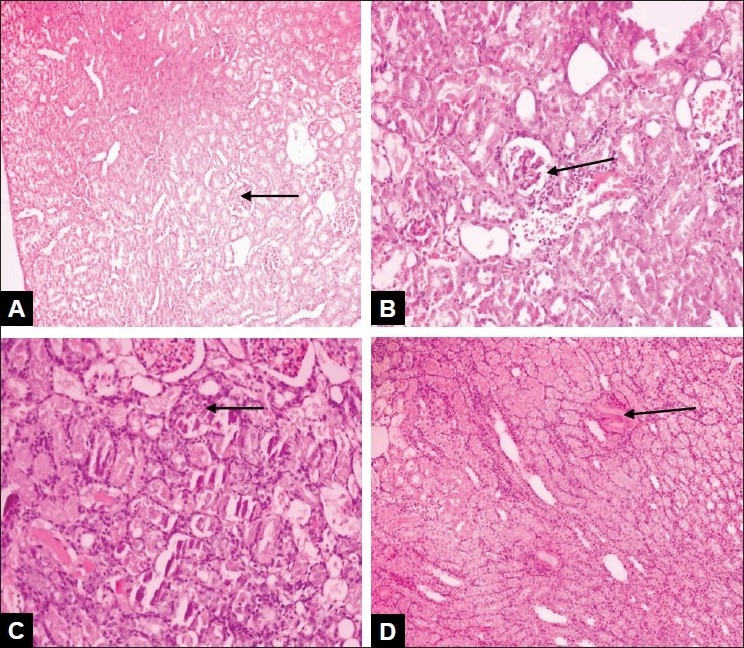
Photomicrographs of rat kidneys from different groups (A): Kidney tissue of control animal with normal glomeruli with an intact bowman's capsule and proximal convoluted capsule and stained with Haematoxylin and Eosin at magnification 40X. (B): Kidney tissue of animal treated with gentamicin with glomerular congestion, inflammatory cells, necrosis and tubular casts and stained with Haematoxylin and Eosin, at magnification 200X. (C): Kidney tissue of leaves extract-treated animals with blood vessel congestion, interstitial edema and tubular casts and stained with Haematoxylin and Eosin, at magnification 800X. (D): Kidney tissue of unripe pods extract-treated animals with normal arrangement of cells and stained with Haematoxylin and Eosin, magnification 100X.

**TABLE 2:** HISTOPATHOLOGICAL FEATURES OF THE KIDNEYS OF RATS OF DIFFERENT TREATMENT GROUPS

Histopathological Feature	Control	Gentamicin treated	Gentamicin and leaves extract treated	Gentamicin and unriped pods extract treated
Glomerular congestion	-	+++	++	+
Blood vessel congestion	-	++	+	--
Interstitial edema	-	++	+	--
Inflammatory cells	-	++	+	--
Necrosis	-	++	--	--
Tubular casts	-	+++	+	+

++ Presence

-- Absence

Our study results showed that the ethanol extract of leaves and unripe pods of *B. purpurea* possessed potent nephroprotective activity. Many Phytochemical reports revealed the presence of flavonoids, carbohydrates, glycosides, tannins, volatile oils, anthocyanidins, lactones and terpenoids[12]. The qualitative phytochemical investigations on the ethanolic extracts also showed positive for flavonoids by ferric chloride, alkaline reagent and Shinoda tests. Further it has been reported that flavonoid constituents of the plant possess antioxidant and hepatoprotective properties[13]. The results of our study suggest that *Bauhinia purpurea* contains constituents having nephroprotective activity. Of the two extracts ethanol extract of unripe pods has significant activity as compared to leaves extract which may be due to the phytoconstituents present in the extract. Further investigations using specific fractions of the extracts can help to isolate and identify potential nephroprotective constituents.
